# The Diagenetic Alterations of Historic Skeletons from the Crown Mines Cemetery, South Africa

**DOI:** 10.3390/biology12030378

**Published:** 2023-02-27

**Authors:** Stacey L. Lander, Margot Hosie, Desiré Brits

**Affiliations:** Human Variation and Identification Research Unit, School of Anatomical Sciences, Faculty of Health Sciences, University of the Witwatersrand, 7 York Road, Parktown, Johannesburg 2193, South Africa

**Keywords:** bone diagenesis, bone histology, bone taphonomy, gold mine cemetery

## Abstract

**Simple Summary:**

The Crown Mines Cemetery, accidently identified in 2010, is another example of the mistreatment of “native” mine cemeteries during Apartheid in South Africa. The large cemetery was intentionally covered/concealed by a mine dump and when later reclaimed, revealed skeletons that were severely flattened, damaged, and fragmented with some bones stained blue. To investigate the individuals buried at the Crown Mine Cemetery, an osteological analysis was attempted; but as the bones were severely damaged and poorly preserved, little information was obtained. The microstructure of bone can sometimes be more helpful in these circumstances. This study therefore investigated the microstructure of the bones buried in the cemetery, firstly, to determine how this specific type of burial environment affected the bones and, secondly, to establish if the microstructure can be used to find out more about these individuals. The results showed that the bones initially began breaking down under normal conditions, yet later may have been exposed to a more acidic environment most likely due to the overlying mine dump and removal thereof. Although, physically, the bones appeared badly damaged, the inner sections of the bones were well-preserved. This can be used for future investigations to find out more about the individuals.

**Abstract:**

Human skeletons associated with early gold mining in Johannesburg, South Africa are investigated. An unmarked cemetery was buried beneath a mine dump which resulted in macroscopically stained and poorly preserved bones. Histological assessments were conducted to understand the postmortem treatment of the remains, determine the extent of bone degradation, and understand how this environment affected the bone’s microstructure. Various diagenetic alterations and the general histological index were assessed using normal and polarized light microscopy of thin anterior midshaft femur sections (n = 50). Degradation was identified in the periosteal and endosteal regions, while the intra-cortical region remained well-preserved. Bacterial bioerosion, microcracks, infiltrations, inclusions, and staining were found throughout the sample. Numerous non-Wedl micro-foci of destruction were observed, filled with exogenous material. The degradation suggested that the remains were buried in neutral soil that was subsequently covered by acidic mine dumps which resulted in a corrosive environment. Although the skeletons were poorly preserved, their histological integrity was more promising, especially the intra-cortical area. This is important for future investigations of archaeological bone, as this area can lead to more accurate descriptions of skeletal assemblages. Targeted sampling of this region could produce promising estimates of age, descriptions of pathology, and biomolecular results, which require further study.

## 1. Introduction

In 2010, a historical Black (“native”) mineworkers’ cemetery (Crown Mines Cemetery), was discovered in the Crown Mines area, south of Johannesburg, South Africa [1]. Cultural material such as cups, plates, and bottles identified near the cemetery was associated with companies in operation during 1890 until 1920 [2]. A fragment of porcelain plate, with the words ‘Robinson Central Deep’ inscribed, further suggested the cemetery was associated with one of the gold mines operating on the Langlaagte farm 224-IQ [2]. Approximately 650 individuals were believed to be buried in this large historical cemetery. The individuals were most likely migrant mine workers, as historical records show a huge influx of people arriving in Langlaagte during the gold rush [1,3].

**Figure 1:**
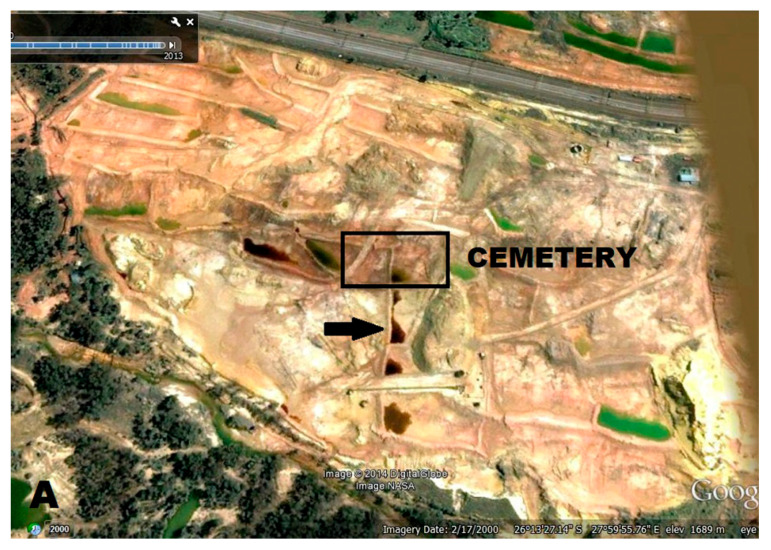

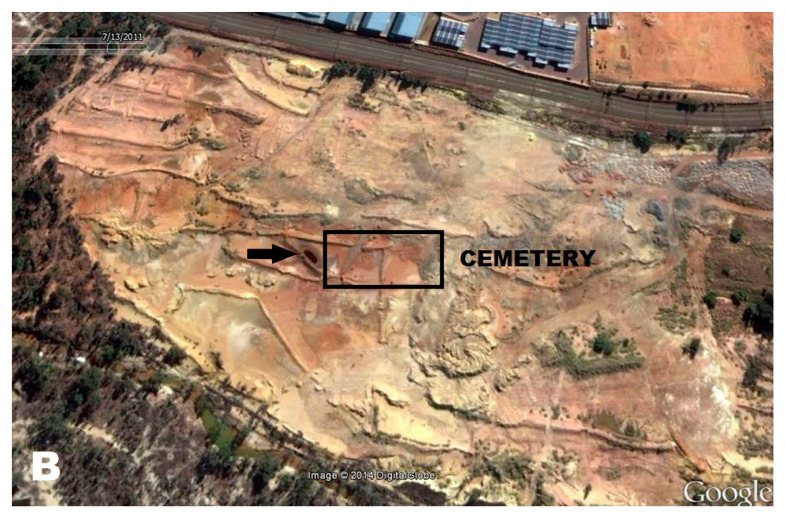
Google Earth images taken in 2000 (**A**) and 2011 (**B**) illustrating the footprint of the mine dump in relation to the historic cemetery (black blocks) and the paddock/s highlighted below or on the left of the cemetery (black arrows).

The cemetery was “accidentally” discovered after the reclaiming of an old gold mine dump (Figure 1), where the mined material, which remained at the site for several years, was removed and reprocessed for gold extraction. This was following the intentional covering/concealment of the cemetery by the mine dump [1]. The removal of the mine waste dump material, as well as additional topsoil, exposed some of the underlying skeletons that were located beneath the waste material. The reclamation process of the mined material most likely involved transporting the dump material to a nearby treatment plant, with repeated watering to erode the dump material away [1,4]. For environmental protection, paddock/s or tailing dams were constructed to minimize the run-off of rainwater and to control for soil erosion [4,5]. The paddock/s shown in Figure 1 are in close proximity to the historical cemetery site. These paddock/s can become acidified when sulfide-bearing material (pyrite) is exposed to oxygen and water, resulting in acid mine drainage [5,6,7]. The acidic water then percolates through the dump into the groundwater systems. Naicker et al. [6] investigated the groundwater systems in the mining district south of Johannesburg and found heavy metal concentrations, high sulfate, and a pH of 3.4. A few bone and soil samples, collected from the historical cemetery, were chemically analyzed using a JOEL JSM-5800 LV scanning electron microscope (SEM) fitted with a Thermo Scientific electron dispersive spectrometer (EDS) [8]. The results indicated increased levels of iron and decreased levels of sodium compared to normal bone levels, and the soil had a pH of 3.5. Iron is a known by-product of acid mine drainage [5,6] and sodium depletion is indicative of bone leaching [9,10]. Repeated watering may have encouraged acid mine drainage to filter through the soil, exposing the skeletal remains to contaminants like sulfates and heavy metals. The continuous leaching may have also caused an acidic burial environment that may have greatly affected the bone’s preservation and its histological integrity.

**Figure 2:**
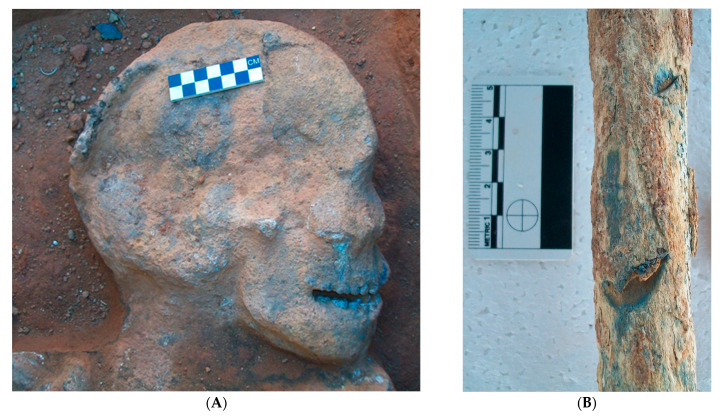
Illustration of (**A**) a skull that appeared ‘cemented’ in situ and (**B**) the blue discoloration of the cortical bone of a femur.

During 2011, exhumations at the mine cemetery began and approximately 100 skeletons were exhumed, and osteological analyses were conducted to establish osteodemographic profiles of the skeletal remains. Unfortunately, the bones were morphologically very poorly preserved, with many of the skeletal elements severely flattened, damaged, and/or fragmented. Several skeletons also appeared to be ‘cemented’ in situ (Figure 2), while the cortical bone of several remains had a blue discoloration (Figure 2). To better understand the postmortem treatment of these remains, investigate the extent of bone degradation, and to establish if histological analyses could be conducted to further describe the skeletal remains, the diagenetic alterations of the historic skeletons from the Crown Mines Cemetery were assessed.

## 2. Materials and Methods

Of the ±100 individuals exhumed; 50 femora were selected, as this was the best preserved element. These remains most likely represented adult individuals based on the epiphysial fusion and dental eruption of the available materials. Based on the in situ assessment of the remains and the fact that the burials were associated with a mine cemetery, they most likely represented males; however, more specific estimates were not possible due to their poor preservation. 

A hacksaw was used to cut a ±5 mm section of bone from the anterior margin of the femoral midshaft (South African Heritage Resources Agency (SAHRA) permit reference: 9/2/228/0096). A manual bone grinding method following stipulations by Maat et al. [11], which is suitable for archaeological bone, was subsequently used to prepare histological sections. To prevent tissue damage and strengthen the fragile sample during grinding, cyanoacrylate glue was used on both the cut bone surfaces and left to harden. Both sides of the sample were then grinded in a rotating motion using P220 waterproof sandpaper and distilled water mixed with detergent. The detergent aided in degreasing the bone sections and as such prevents oily microscopic images. The bone sections were grinded until it was opaque, washed, and left to dry. The dry section was then mounted onto a glass slide and cover-slipped. This method allowed for the quick and easy preparation of histological samples at low cost, which produced high quality images [11,12].

Each bone section was divided into three regions: periosteal (outer surface), intra-cortical (mesosteal or central portion) and endosteal (inner surface). Each region of the thin femoral sections was subsequently analyzed by the first author, SL Lander using normal and polarized light microscopy at 5×, 50×, 100×, and 400× magnifications. Six diagenetic alterations were assessed according to Jans et al. [13]: bioerosion, microcracks, birefringence, inclusions, infiltrations, and staining. The general histological destruction of each bone section was also evaluated following stipulations by Hollund et al. [14] to provide a summary of its histological preservation. 

### 2.1. Bioerosion

**Figure 3:**
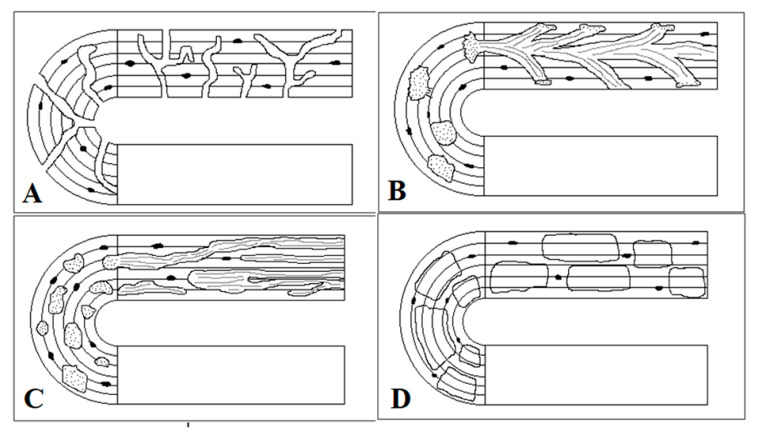
Schematic depiction of the different types of micro-foci of destruction (MFD) in relation to the Haversian canal with depictions in the transverse section (semi-circular part of image) and longitudinal section (horizontal part of image). (**A**)—Wedl, (**B**)—Budded, (**C**)—Linear longitudinal, (**D**)—Lamellate (Images redrawn and adapted from Hackett 1981 [15]).

Bioerosion is the chemical breakdown of bone by microorganisms that results in microscopic tunnels, commonly referred to as micro-foci of destruction (MFD) after Hackett [15]. The tunnels occur most frequently in the sub-periosteal and sub-endosteal areas of the bone, avoiding the periosteal and endosteal fringes despite these surfaces being in contact with the soil [16,17]. The microorganisms most commonly responsible for MFD are fungi and bacteria. They are categorized according to their specific pattern of tunnelling and five different types are identified and described in the literature. They are Wedl, linear longitudinal, budded, lamellate, and Hackett [15,18,19,20]. These are represented in Figure 3, except for the Hackett MFD, due to its location. Wedl MFD is of fungal origin with two types described by Trueman and Martill [19]. Wedl Type 1 is more common and is found within the periosteum and endosteum. Wedl Type 2 is exclusively associated with the intra-cortical bone, extending from the Haversian canals. Hackett MFD is also of fungal origin but is mainly concentrated superficially, radiating into the bone’s surface [18,20]. The other non-Wedl types of MFD described (linear longitudinal, budded, and lamellate) are of bacterial origin and they are the most common form of microbial tunnelling in archaeological human bone [16,21]. They occur most frequently around the Haversian canal, filling the Haversian system [15,22,23,24], as well as emerging from osteocyte lacunae [21,25]. Lamellate tunnels, in particular, can also be abundantly found towards the periosteal surface.

The five types of MFD were qualitatively assessed for each bone section. They were elaborated upon according to their shape (i.e., long, elongated, or round) and their possible origin and distribution. The presence of cuffing, often related to bacterial MFD, was also evaluated. A cuff can be produced during tunnel formation due to mineral redeposition or remineralization of the exposed surface areas of the microscopic tunnel [15].

### 2.2. Microcracks

Two types of microcracks can occur in archaeological bone and care should be taken when distinguishing them from one another. There are artefact cracks or microcracks due to processing and handling of the bone sample, as well as those directly related to bone diagenesis. Diagenetically linked microcracks have been linked to the loss of collagen which causes shrinkage, the deposition of calcium carbonates which disintegrates the microstructure, and remineralization processes resulting from microbial activity [13,14]. The microcracks were qualitatively described and not quantified because of the difficulty to distinguish the diagenetically associated microcracks from those resulting from the sample preparation. The microcracks were therefore described by recording the areas of the bone (i.e., interstitial lamellae) in which they were noted. 

### 2.3. Birefringence

The loss of bone collagen, as well as other structural properties like the hydroxyapatite crystals, can be assessed using birefringence. It investigates the quality of the diagenetically altered bone by using polarized light microscopy. Under normal circumstances, a specific pattern of alternating bright and dark bands is observed due to the orientation and density of the collagen fibers present. Bone that is positively birefringent therefore has collagen fibers that are more transversely orientated or increased in density compared to their adjacent collagen fibers. In comparison, a negative birefringence indicates the deterioration of collagen and/or the loss of orientation of the hydroxyapatite crystals [14,26]. Using a polarized lens, the intensity of birefringence was evaluated for the three regions of bone, to assess the integrity of the bone collagen. Each region was then categorized between 0 and 1 in line with work by Hollund et al. [14] with 0 indicating no birefringence, 0.5 indicating reduced birefringence, and 1 indicating perfect birefringence. Perfect birefringence was indicative of the observer’s ability to see alternating bright and dark bands of lamellae characterized by the Maltese cross pattern across the Haversian systems, whereas this was absent with no birefringence. 

### 2.4. Inclusions, Infiltrations, and Staining

Garland [27] defines inclusions as “the presence of externally derived material lying within the available bone spaces; namely Haversian canals, osteocyte lacunae and canaliculi” [27] (p. 226). Examples of such material include sand, fungal cells, hyphae, rhizomorphs, bacteria, insect parts, and framboidal pyrite [13,27]. In contrast, infiltrations are defined as “the presence of unrelated material within the bone substance itself” [27] (pp. 226). When observed under low magnification, infiltrations have a granular appearance; while it looks like the bone matrix has been replaced by non-osseous derived material, when observed under high magnification [27]. Stained archaeological bone is also considered a form of infiltration [13,14], but to prevent confusion it is referred to as “staining” hence forth. 

Using the definitions given by Garland [27], the inclusions were qualitatively identified according to their location (i.e., Haversian canal) and color (i.e., blue, or black). Similarly, infiltrations were recorded according to their location and color, as well as their shape (i.e., round, thin or long). Staining was also described by location and color.

### 2.5. General Histological Index (GHI)

To quantify the extent of bone diagenesis in each femoral section, the GHI developed by Hollund et al. [14] was used to summarize the amount of altered versus unaltered bone present. This included bioerosion, microcracking, staining, and overall generalized destruction of the bone sections. Using Table 2 of Hollund et al. [14] (p. 541), the descriptions given in conjunction with the approximate percentage of intact bone observed, the three regions of bone were given a score of 0–5, where the lower the score the more diagenetically altered the section was. This provided the overall preservation of each bone section and its different regions. 

For consistency, data were collected by the same observer, twice. However, 5% of the bone samples were analyzed for interobserver repeatability testing using a Weighted Cohen’s Kappa, which indicated moderate agreement between observers (κ = 0.459).

## 3. Results

**Figure 4:**
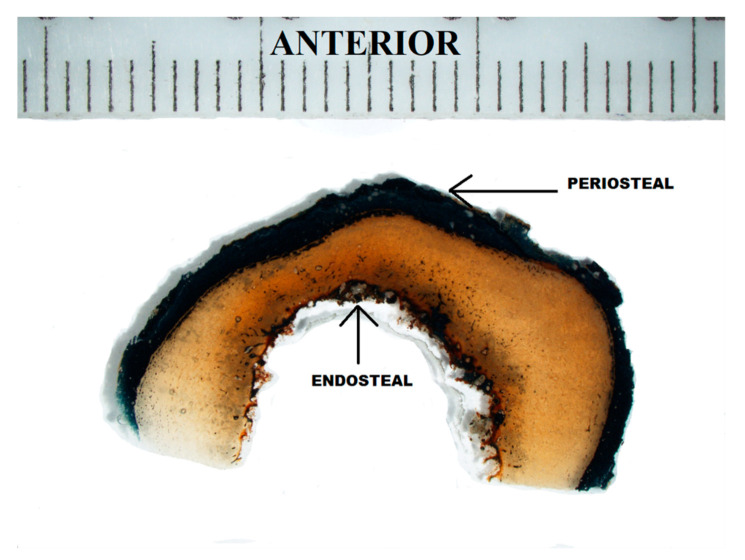
Cross-section from the anterior femoral midshaft showing the diagenetically altered periosteal and endosteal regions of the bone sample (1 mm ruler at 5× magnification).

**Figure 5:**
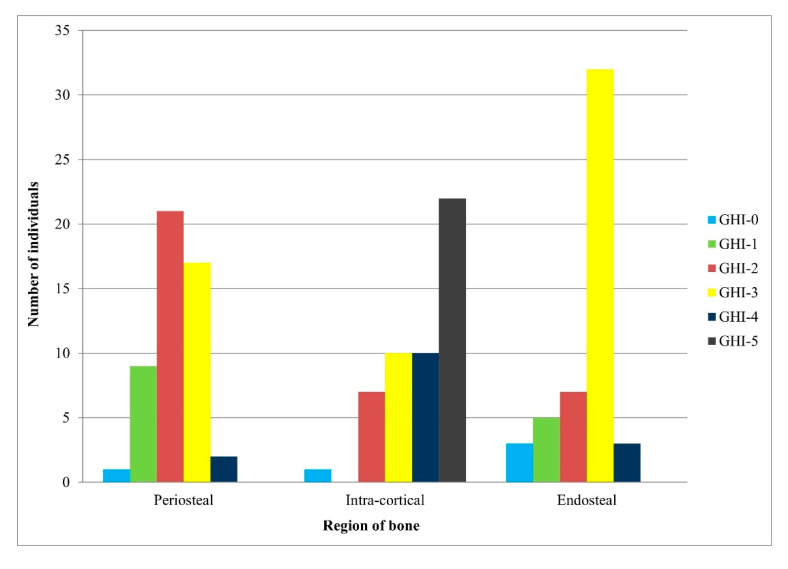
The general histological destruction for the periosteal, intra-cortical, and endosteal regions of bone, quantified using the General Histological Index (GHI) developed by Hollund et al. [14].

Diagenetic alterations were mainly identified at the periosteal and endosteal surfaces of the bone, leaving the intra-cortical regions relatively well-preserved (Figure 4 and Figure 5). The periosteal areas had approximately 16–49% intact bone present (GHI = 2), whereas the endosteal areas had about 50–84% intact bone present (GHI = 3) (Figure 5). The well-preserved intra-cortical regions had predominantly 95–100% intact bone present (GHI = 5) (Figure 5), similar to normal bone. 

The majority of the bone degradation toward the periosteum was due to non-Wedl MFD that was filled with blue exogenous material (inclusions). Due to the difficulty in qualitatively and accurately distinguishing the specific type of MFD present (budded, linear longitudinal or lamellate); their overall shape, location, and the presence of cuffing were used to identify them as non-Wedl MFD. The coalescent nature of the non-Wedl MFD in this area resulted in the blue discoloration observed macroscopically (Figure 2 and Figure 4). Due to the poor preservation of the skeletal remains (i.e., periosteal bone degradation), many of the periosteal fringes were lost postmortem and during bone sampling/preparation.

**Figure 6:**
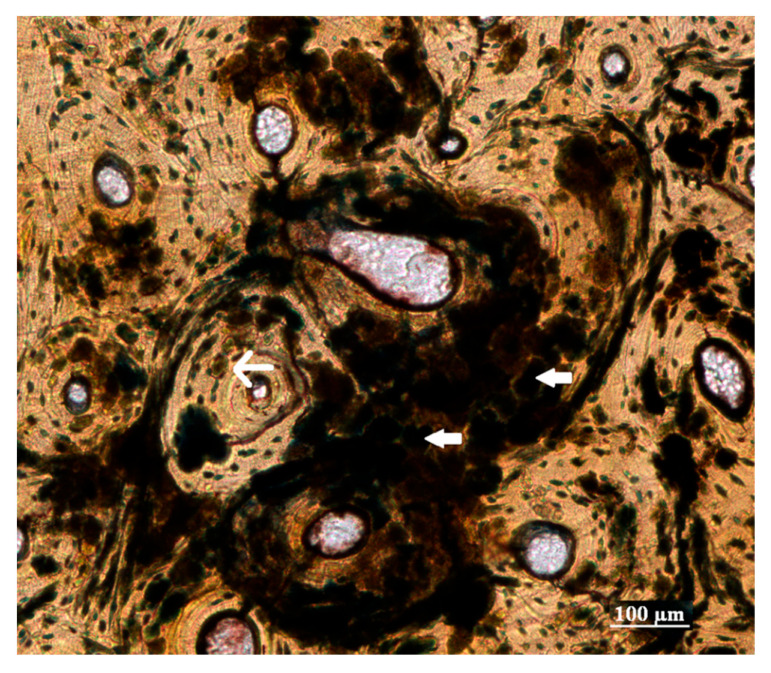
Non-Wedl MFD, spherical in shape and blue-green or brown in color, filled the Haversian systems and enlarged the osteocyte lacunae in the intra-cortical region (400× magnification) (thick white arrows). The thin white arrow indicates cuffing.

**Figure 7:**
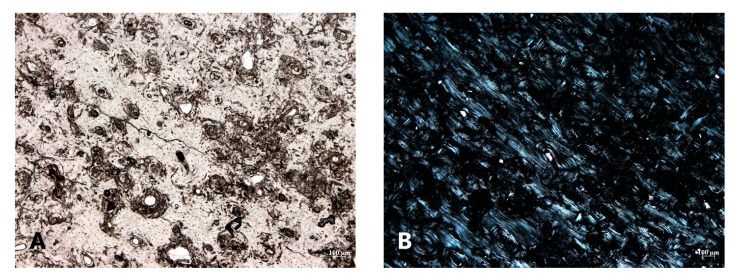

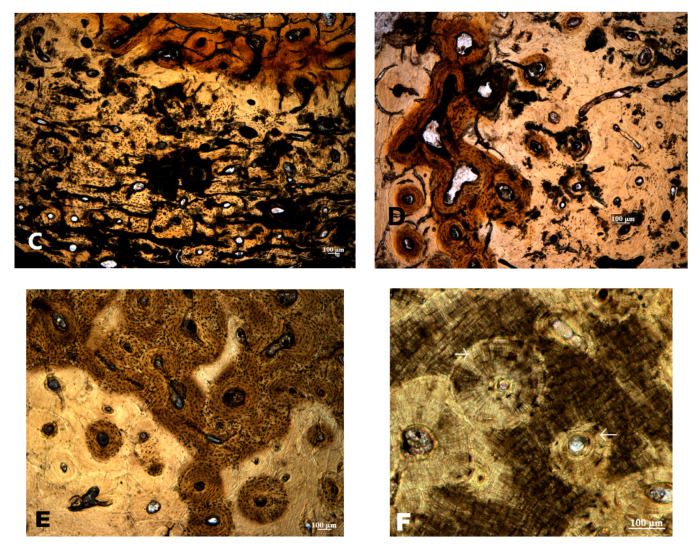
Diagenetic alterations observed in the bone. (**A**,**B**)—An illustration of many non-Wedl MFD in the intra-cortical region under normal and polarized light (50× magnification); (**C**)—Numerous non-Wedl MFD in the intra-cortical region with orange staining toward the endosteum (50× magnification); (**D**,**E**)—Black inclusions in selective osteocyte lacunae associated with orange or brown staining in the intra-cortical region (100× magnification); (**F**)—Black inclusions (white arrow) in some osteocyte lacunae and canaliculi (400× magnification).

Non-Wedl MFD was commonly found throughout the entire bone section. Not only was it identified toward the periosteal surfaces, where it was predominantly noted, but it was also present in the intra-cortical and endosteal areas of some bone sections (Figure 6 and Figure 7). It appeared to originate from the Haversian canals and the osteocyte lacunae. This resulted in the “filling” of the Haversian system with groupings of MFD in the concentric lamellae due to enlarged osteocyte lacunae. In the periosteal regions in particular, the MFD appeared to follow the microstructure of the bone by displaying parallel rows, as it arranged itself within the circumferential lamellae. Overall, its shape was spherical and varied in size, with the majority of it filled with exogenous material (or inclusions) that displayed a blue, green, brown, orange, and red color. The filled MFD was not birefringent (opaque); however, when less inclusions were present, cuffing was observed (Figure 6). The periosteal regions that contained coalesced non-Wedl MFD were also not birefringent, while the intra-cortical and endosteal regions that contained some MFD had reduced birefringence (Figure 7). It is important to note that the observation of these inclusions inside the MFD indicate that their inclusion postdates the bacterial degradation process.

In addition to the inclusions identified within the non-Wedl MFD, inclusions were also found in the medullary cavity (spongy bone), Volkmann’s canals, Haversian canals, osteocyte lacunae, and canaliculi (Figure 6 and Figure 7). The majority of the exogenous material present in these particular bone spaces was black in color; however, red, orange, blue, and green inclusions were also identified. The distribution and location of the inclusions varied between the bone samples. Inclusions in selective osteocyte lacunae of one individual radiated to other osteocyte lacunae via their canaliculi, whereas in other selective osteocyte lacunae, inclusions appeared to be associated with orange or brown stained bone (Figure 7). In the medullary cavity, round, black inclusions were also found in close proximity to a thin, orange-stained rim of bone, while other inclusions not associated with stained bone were also identified. It is important to note that although some smaller bone spaces may have appeared to have black inclusions present (i.e., lacunae or canaliculi), air can also cause dark artefacts.

**Figure 8:**
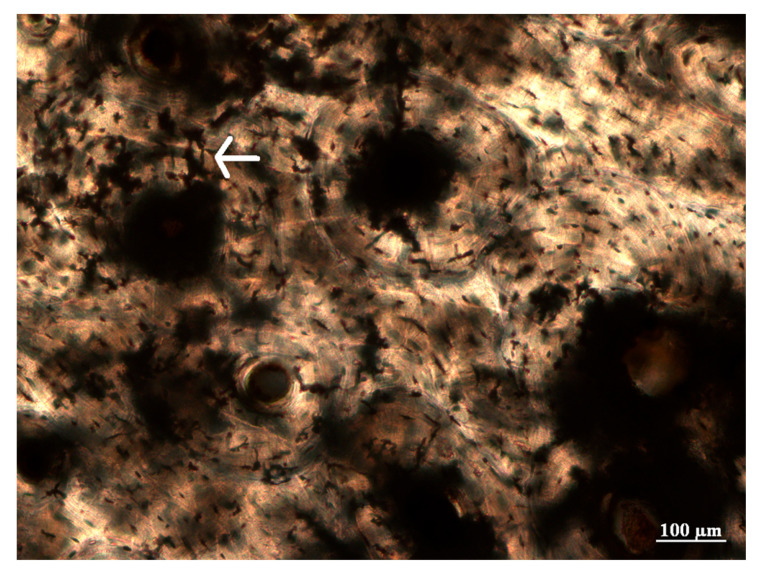
Infiltrations in the intra-cortical region that were long and thin in shape and displayed a dark brown or black color (400× magnification).

Microcracks, infiltrations, and staining were also identified in the archaeological bone. Microcracks were found in both concentric and interstitial lamellae and often radiated from the Haversian canals. Infiltrations also radiated from the Haversian canals but also from osteocyte lacunae in some bone samples (Figure 8). They were long and thin in shape, with variable thicknesses, appeared solid at high magnification, were not birefringent (opaque), and displayed a dark brown/black color. As mentioned previously, orange, and brown staining was commonly identified in the archaeological bone (Figure 7), but green and red staining was also observed. Orange and red staining was predominantly identified toward the periosteal and endosteal surfaces, while one bone sample had green staining and a few others had brown staining in the intra-cortical region (Figure 7). The stained areas of bone had perfect birefringence.

## 4. Discussion

Regrettably, little is known about the life and post-life history of the individuals buried at the Crown Mine Cemetery and this is in part due to the lack of maintenance, intentional concealment, and erasure of “native” mine cemeteries during Apartheid in South Africa [1]. By studying the diagenetic alterations of the bone, we are not only informed about the preservation of the skeletal material, but also the postmortem treatment of these individuals. The patterns of diagenetic alterations observed are also consistent with decedents buried after death in aerobic soil but later covered by mine waste tailings and consequently affected by the treatment of acid water (paddocks) during the reclamation process [1]. Although this study aimed to assess the preservation of the skeletal remains from the Crown Mine Cemetery, it was important to focus our efforts on the best preserved skeletal element to ensure that samples were selected from the same bone (femur) and at the same site (midshaft). It is therefore important to note that the preservation described is related to the femur only and might not reflect the preservation of the other skeletal elements.

Various factors can alter the integrity of skeletal remains; however, these processes are not yet well understood [28]. Diagenetic bone alterations are often not macroscopically visible [28] and although morphologically the Crown Mines’ skeletons were poorly preserved, their histological integrity remained relatively intact. The majority of the bone specimens had an intra-cortical region that was well-preserved (95–100% intact bone, GHI = 5), with diagenetic alterations mainly identified toward the periosteum (16–49% intact bone, GHI = 2) and endosteum (50–84% intact bone, GHI = 3). This is not uncommon in archaeological bone and a similar pattern of diagenesis was observed in skeletal material from medieval contexts [29], an AD 100-300 Roman burial site located in Castricum, the Netherlands [14] and medieval sites from Castlegate and Hungate in York, UK [30]. Considering that the periosteal surface of the bone is the initial point of contact, it is not surprising that this region was more diagenetically altered in this study, compared with the endosteal and intra-cortical regions that were more protected from external factors. 

Factors affecting bone degradation, thus resulting in the diagenetic alterations observed at the Crown Mines Cemetery, include burial environment and early postmortem events [16,20,21,28,31,32]. Like the Crown Mine bone, microbial bioerosion is the most common form of diagenetic alteration of archaeological bone [32]. It normally occurs during the early stages of decomposition, mostly confined to the first decade after death and primarily caused by endogenous gut bacteria and is generally found within remains from chemically benign (neutral pH) environments [16,21,25,28,31]. For fully interred human remains in benign soils, microbial attack or its microbial features are also easier to identify because of better mineral preservation [31]. In more corrosive soils (low pH), however, catastrophic mineral dissolution of the bone will result in the long-term. For the Crown Mines Cemetery, the chemical analyses indicated that the soil surrounding the bone had a pH of 3.5, but the bone itself showed good histological integrity, especially for the intra-cortical region. This does not support a long-term acidic environment, or entirely aquatic deposition. 

On the other hand, the macroscopic blue colored cortical bone observed in the periosteal surfaces of many Crown Mine bones was likely due to non-Wedl MFD filled with blue-green exogenous material. Macroscopic bone staining is not uncommon in archaeological remains and has been linked to acid metabolites from saprophytic microorganisms [33], cyanobacteria and algae [18,28], as well as impurities of transitional trace metal ions like chromium, manganese, iron, cobalt, nickel, and copper [10,34,35]. Acid mine drainage in the south of Johannesburg is known to have high concentrations of iron, aluminum, manganese, and sulfate, as well as low concentrations of toxic heavy metals [6,7]. The exact contaminants present in the paddock water that was near the skeletal remains at the Crown Mines Cemetery are unknown, but the water color observed in the Google Earth images appeared to be brown and green, which is suggestive of a heavy iron load and a metal salt precipitate, respectively. The chemical analyses also indicated a high level of iron in the bone samples [8]. In addition to this, the histological orange and brown-stained bone identified in this study, has been previously associated with iron oxides [14,18,35]. Evidence of framboidal pyrite crystals in particular, specifically associated with orange-stained bone in this study, was also identified. Hollund et al. [14] suggested that this is indicative of FeS2 (iron sulfide) being partially oxidized to form iron oxides and is commonly found in gold mine tailings [5]. The blue-green discoloration can also be associated with cyanobacteria infiltrating bone exposed to sunlight [28]. This along with the presence of framboidal pyrite could also indicate degradation in acidic and/or waterlogged conditions [28]. However, the presence of non-Wedl MFD, associated with terrestrial degradation, could point to some of the remains being waterlogged for a period of time [1]. 

Bone diagenesis within the Crown Mines’ skeletons therefore suggests exposure to both benign and corrosive soil environments [1,28]. The initial benign soil would have encouraged non-Wedl bioerosion and the microscopic red or brown staining, as well as the possible organic infiltrations and inclusions due to humic material in the soil [13,28,36,37]. The subsequent deposit of mine dump material may have crushed and deformed the skeletal remains beneath it, and as they contain rock, clay, chemical contaminants, and no organic material, it can also result in acid mine drainage due to the leaching of metal sulfides [1]. Leaching of the bone may have occurred because of the sandy soil type present in the area [1,8], which allows for large amounts of water to pass through the soil rapidly [38]. After that, the reclamation process may have drastically increased the acidity of the soil due to the paddocks or tailing dams which were near the buried remains [1]. The corrosive soil may have caused severe macroscopic damage of the bones (brittle and deformed), possibly degrading their periosteal surfaces, resulting in collagen loss and fluorescent inclusions present in the tunnels of the non-Wedl MFD. Although the burial environment at the time of exhumation of the Crown Mines’ skeletons was acidic, the histological analysis of the bone suggests that the acidity may have only occurred in the latter years of interment. 

The value of investigating archaeological bone histology is continuously realized [32,39,40,41,42,43,44,45]. Considering that the morphological structure of the bone was severely degraded in the Crown Mines’ skeletons, which significantly limited the accuracy of estimations included in the biological profile, a well-preserved intra-cortical region was still present. If researchers are willing to forego the destructive nature of histological analyses, to not compromise on the quality of the diagenetic data collected [46], future investigations that utilize the intra-cortical region can lead to more accurate descriptions of skeletal assemblages, especially for research related to age estimation and descriptions of bone pathology. Furthermore, targeted sampling of this particular area of bone could produce good biomolecular yields for DNA preservation [30]. 

## 5. Conclusions

The Crown Mines Cemetery is yet another example of the mistreatment of “native” mine cemeteries during Apartheid in South Africa. The historical significance of the individuals buried there was completely disregarded when they were later intentionally covered/concealed by a mine dump. The diagenetic alterations identified in the skeletal remains buried in this cemetery suggests exposure to an acidic environment caused by the acid mine drainage and related mining activities. This most likely resulted in the blue discoloration of their cortical bone surfaces, as well as the macroscopically poor preservation of the bones. The intra-cortical region of bone did however show promising histological integrity. This may be due to the bone’s limited exposure to the acidic burial environment, as the results suggest this may have only been for a limited time period occurring in the later years of inhumation. This indicates that the intra-cortical regions could be explored during future research to more accurately describe the individuals associated with the mine. This may also have added value in future investigations of archaeological bone histology (when presented with a similar burial environment) because targeted sampling of the intra-cortical region, in particular, could produce promising estimates of age, descriptions of bone pathology, and biomolecular results. 

## Data Availability

The data collected during this study are available from the corresponding author upon reasonable request, subject to the approval from the South African Heritage Resources Agency (SAHRA).
